# Supplementary material for: Isolation of a Novel Anti-Diabetic α-Glucosidase Oligo-Peptide Inhibitor from Fermented Rice Bran
Source: Foods. 2023 Jan 1;12(1):183. doi: 10.3390/foods12010183 (PMC9818066; doi:10.3390/foods12010183)
Supplement: Supplementary file 1 [file foods-12-00183-s001.zip › foods-1979875-supplementary.pdf]

Isolation of a novel anti-diabetic α-glucosidase oligo-peptide inhibitor from fermented rice bran

Jingfei Hu[a,b], Xiaohua Lai[a,b], Huanyu Wang[a,b], Nanhai Weng[a,b], Jing Lu[a,b,*], Mingsheng Lyu[a,b], Shujun Wang[a,b,*]

[a]Jiangsu Key Laboratory of Marine Bioresources and Environment/Jiangsu Key Laboratory of Marine Biotechnology, Jiangsu Ocean University, Lianyungang 222005, China

[b] Co-Innovation Center of Jiangsu Marine Bio-industry Technology, Jiangsu Ocean University, Lianyungang 222005, China

* **Correspondence.** J. Lu and S. Wang, Jiangsu Key Laboratory of Marine Bioresources and Environment, Jiangsu Ocean University, Lianyungang, 222005, PR China. E-mail addresses: jinglu@jou.edu.cn, sjwang@jou.edu.cn

The collecting of ultrofilter was detected by LC-MS/MS. The result as Figure S1.

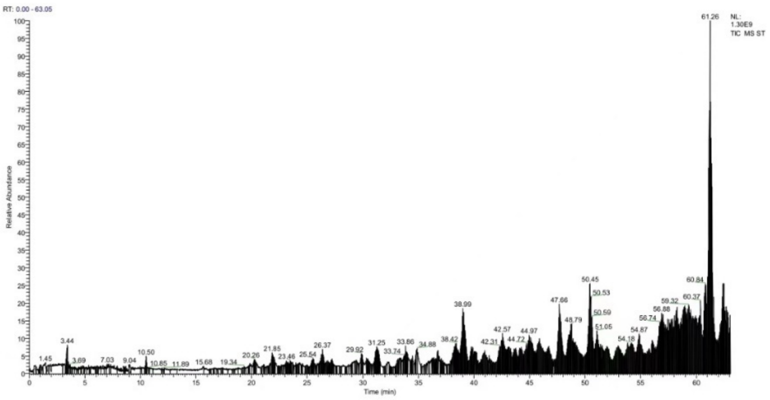

Figure S1. LC-MS/MS profile for rice bran peptide

The GLLGY was synthesis and purified by HPLC (Figure S2).

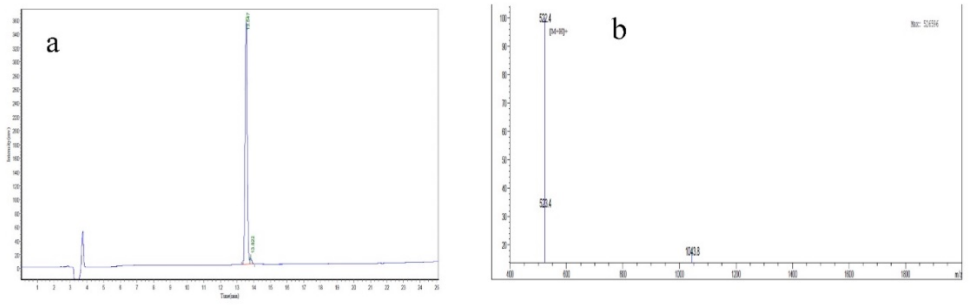

Figure S2. **(a)** LC-MS/MS chromatogram of GLLGY obtained from preparative RP-HPLC. **(b)** ESI-MS/MS spectrum of the most intense peptide at m/z 522.292.
